# Headache and Psychological Comorbidities: An Appraisal of the Evidence

**DOI:** 10.3390/jcm12072683

**Published:** 2023-04-04

**Authors:** Ishaq Abu-Arafeh

**Affiliations:** Paediatric Neurosciences Unit, Royal Hospital for Children, Glasgow G51 4TF, UK; ishaq.abu-arafeh@ggc.scot.nhs.uk

**Keywords:** adolescents, anxiety, children, depression, headache, migraine, psychological disorders

## Abstract

Background: It has been observed that there is a higher-than-expected risk of anxiety and depression in children with chronic headache and also an increased risk for the persistence of headache in patients with anxiety and depression. Objectives: This review aims to identify and assess the relationships between primary headache disorders and comorbid emotional and psychological disorders. Methods: A targeted review of the literature was carried out. Results: The associations between the disorders are more pronounced in clinic patients, who may represent the severe end of the headache spectrum, but less clear in patients who were identified in population-based studies and who may represent the “average” child with headache or the “average” child with psychological disorders. Conclusions: Understanding this bidirectional association of comorbid disorders is of great importance to offering a holistic biopsychosocial approach to the management of headache disorders in children and adolescents and in addressing the risks for and the co-existence of psychological comorbidities.

## 1. Introduction

Primary headache disorders are common and affect people of all ages and all socioeconomic backgrounds. Primary headaches are reported by people all over the world regardless of race or ethnic origin [1,2]. Furthermore, headache may occur in people with no additional health problems as well as in those with other medical illnesses. Headaches can also affect people with mental health disorders and those with personality disorders as well as those with no mental health issues.

Primary headache disorders are common in children and adults. Around 60% of children and adolescents experience at least three episodes of headache per year, and around 8% suffer from migraine [1]. Episodic tension-type headache (ETTH) affects around 20% of children and adolescents [2].

Despite their biological and complex genetic aetiology and pathogenesis, primary headaches follow the biopsychosocial model in their presentation, trigger and relieving factors and also in their response to pharmacological treatment and non-pharmacological therapies. The biological basis of headache disorders and genetic predisposition were demonstrated in several family and twin studies [3]. The genetic basis of headache disorders, and migraine, in particular, is a complex polygenic inheritance. Therefore, the disease expression can be influenced by external environmental factors, internal physiological changes and also psychological influences, among other factors.

The pathogenesis of primary headaches, and migraine, in particular, involves an extensive neuronal network of the brain, several neurotransmitters and polypeptides that have receptors across the brain with variable functional responses. Dopamine, GABA and serotonin are among other neurotransmitters that have been shown to play parts in the pathogenesis of migraine and depression [4,5].

The relationship between headache and psychological comorbidities is probably bi-directional, as shown in a prospective study of 1007 young adults (21–30 years of age) in the USA. The subjects were interviewed at the time of recruitment and 3.5 years later. The estimated relative risk for major depression associated with prior migraine was 3.2 (95% CI 2.3–4.6), and the adjusted relative risk for migraine associated with prior major depression was 3.1 (95% CI 2.0–5.0) [6]. Similar results were also found in a retrospective study of 1284 people (25–55 years of age) in which a lifetime prevalence of major depression was approximately three times higher in persons with migraine and in persons with severe headaches compared with controls [7]. Significant bidirectional relationships were observed between major depression and migraine, with migraine strongly predicting first-onset depression (hazard ratio = 3.6) and, to a lesser extent, depression predicting first-onset migraine (hazard ratio = 1.6) [7].

Children with frequent or chronic headaches are at particular risk of psychological comorbidities that may overshadow the original complaint or exacerbate the headache disorder, rendering treatment more challenging or, at times, ineffective. Stress is often reported to be the most common trigger factor in children [8]. Such a relationship between stress and headache attacks may be confused with a causal relationship.

Several studies have also shown an increased prevalence of anxiety, depression and other pain conditions in mothers of children with headaches, suggesting maternal factors as risk factors for headaches in children [9].

Although migraine and tension-type headaches are the most common primary headaches in the wider childhood population, chronic daily headaches and particularly chronic migraine and medication overuse headache, are increasingly the leading causes for referral and assessment of adolescents with headaches in specialist centres [10,11].

Chronic headaches can have a disabling impact on the quality of life of children and adolescents, with adverse outcomes on health, education, social functioning and family life [12,13]. Previous research has shown that children and young people with certain psychological traits and social characteristics may be at a higher risk of developing chronic headaches. A number of studies which are discussed in more detail below, present evidence that young patients with chronic headache are more likely to suffer from psychological disorders such as depression, anxiety and behavioural difficulties.

Understanding the intricate relationship between headache and psychological disorders may help in the successful treatment of chronic headache in children and adolescents. Therefore, future research may benefit from using integrated research methodologies with a focus on psychological factors in the treatment of chronic headache in young people.

## 2. Pitfalls in The Study of Headache and Psychological Comorbidities in Children

The literature on the psychological comorbidities in children with headache can be difficult to interpret, and conclusions can be difficult to reach due to variations in methodology, settings, children’s ages and the population base of different publications. In order to better understand the published studies, the following important points are highlighted:

(1) Population-based studies provide data on an unselected group of children and, potentially, may provide conclusions on comorbid disorders that are more generalisable in children with headache. On the other hand, studies on clinic populations provide important information and insight into these groups of patients but cannot be generalised due to unavoidable selection bias.
(2) Different studies may report on different age groups from within the childhood population. Preschool-age children may have different issues than school children or adolescents. Psychiatric comorbidities may take different clinical presentations at different age groups.
(3) Children with different types of headache disorders may need to be studied separately. A distinction between children with episodic migraine (EM), chronic migraine (CM), episodic tension-type headache (ETTH), chronic tension-type headache (CTTH), other chronic daily headaches and mixed headaches should be made, as they may have a different predisposition to comorbidities or future complicating disorders.
(4) Children with a long history of headache disorder and those with a high frequency of headache attacks may constitute a different group than children with a short history and infrequent attacks of headache.
(5) The use of the International Classification of Headache Disorders and acceptable definitions and screening tools for different psychological and personality disorders, such as anxiety and depression, is important in order to compare the results of different studies.

## 3. Screening for Psychological Comorbidities in Children and Adolescents with Headaches

A meta-analysis of published studies on children and adolescents with headaches showed a higher incidence of psychopathological characteristics in those with migraine and tension-type headaches than in control groups [14]. The meta-analysis included eight studies using the Child Behavior Checklist for internalising (CBCLi) and externalising behaviour (CBCLe). Unfortunately, the meta-analysis was unable to distinguish between children recruited from clinic-based studies and population-based studies. It has also been unable to distinguish between those participants with episodic (migraine or tension-type) headache and chronic (migraine or tension-type) headache. Hence, it was unable to identify any predicting factors for the association [14]. However, it was useful in confirming the presence of comorbidities and in the usefulness of the CBCL as a useful screening tool.

A recent controlled study of 112 adolescents with migraine and 122 control adolescents (13–18 years of age) showed a higher incidence of anxiety as identified by the Beck Anxiety Inventory. The diagnosis of migraine was based on the International Classification of Headache Disorders (ICHD-3). Control adolescents were more likely to have a non-depressive score than those with migraine on the Children’s Depression Inventory. Adolescents with migraine who also had moderate or severe anxiety or depression experienced more severe and more frequent migraine attacks [15].

## 4. Children with Episodic Headache

### 4.1. Population-Based Studies on Episodic Headache and Psychological Comorbidities

Few population-based studies have looked at the prevalence of psychological disorders in children with EM and ETTH. Some studies reported on the parental description of the child’s personality and behaviour or children’s self-reporting of their own perception of their personality traits [8,9]. Other studies may have used a validated checklist, questionnaire, face-to-face interviews or formal psychiatric assessment. Population-based studies give balanced views of the children’s psychological comorbidities, and they seem to show little or no differences between children with episodic headache and those with no headache. Furthermore, there seem to be no significant differences between children with EM and those with ETTH [16].

A longitudinal epidemiological study of around 800 children and adolescents between seven and eighteen years of age who were followed up for at least seven years showed headache (unspecified headache disorder) was approximately twice as common in depressed adolescents compared with the non-depressed. Adolescents who had no history of chronic impairing headaches but have current major depression had nearly a tenfold increased risk of developing headaches at some time during the following seven years [17].

In a study of 12-year-old Finnish schoolchildren the prevalence of ETTH was 12.2%. Up to 17% of children with ETTH had more than two depressive symptoms compared to only 6% of children without headaches. Furthermore, 1.5% fulfilled the criteria for major depression as compared to none in children without headaches [18].

Behavioural attributes of children with headaches may be evident from a very early age. A study of headaches in preschool-age children (under five years) found an increased risk of temper tantrums of 1.4-fold in those with frequent headaches compared to children with infrequent or without headaches. There was also an increased risk of behavioural problems, diurnal enuresis and problems with attention. In this study, there was no distinction between EM and ETTH, and there was no quantification of the frequency of headaches or the quality of life [19].

A large population-based study of migraine in schoolchildren (5–15 years) found no differences in the social background between children with migraine and a matched control group of children without headache [8]. The same study also showed that 71% of children with EM (described by their parents or describing themselves) as happy compared to 75% of the control group. Eight per cent of children with migraine were described as anxious, 5% as sensitive and 16% as moody compared to 4%, 3% and 16% of the control group, respectively. The differences were not statistically significant [8].

A study of a birth cohort of over 17,000 children born in Bristol, UK, in 1958 was followed up regularly for symptom collection at ages 7, 11, 16, 23 and 33 years [20]. In 1991 at a cohort age of 33 years, 11,407 (69%) were interviewed. Those with headaches at age 11 years were more likely to have mothers with chronic illness or a family member with mental illness. Those with frequent headaches during childhood had an increased risk for multiple physical symptoms (Odds Ratio 1.75 (95% CI 1.46–2.10) and psychiatric comorbidity (Odds Ratio 1.41 (95% CI 1.20–1.66). The increased risk for comorbidity becomes apparent in adult life [20].

The presence of other pain disorders and high-frequency headaches may be associated with an increased risk of anxiety and depression, as shown in a study of schoolchildren (13–19 years) in Sweden, in which two-thirds of children with headache (unspecified) had at least one other frequent pain disorder of back pain, muscle pain or abdominal pain. Children with frequent headaches (occurring on at least one day/week) also had a higher score for anxiety and depressive symptoms [16]. Another Swedish study of a random sample of schoolchildren with headaches (identified from a large prevalence study) showed different results [21]. In this study using the Child Behaviour Check List (CBCL) questionnaire, children with migraine had a higher level of somatic complaints and a higher mean score for internalising symptoms than control children, but not in children with tension-type headaches. Internalising symptoms and withdrawal behaviour did not differ in children with migraine, TTH and children without headache [21].

Stressful life experiences were more often reported by schoolchildren with any type of headache than children without headaches. Children with headaches reported stress in relation to dissatisfaction with a job, excessive school demands, a lack of social recognition, social isolation and chronic worries. Stress was more prominent in children with migraine or those with migraine and TTH than in those with ETTH alone, suggesting that migraine may have a specific risk for association with stress [22].

Symptoms of hyperactivity-impulsivity are more likely to occur in children with migraine (relative risk 2.6 (95% CI 1.4–3.2) than in children without headache. However, inattention and full ADHD did not predict any type of headache [23].

A review of all studies published between 1980 and 2007 on psychological comorbidities in children with migraine did not provide conclusive findings for the comorbidity of migraine, anxiety and depression in children. The studies, however, lacked homogeneity, and only three of the 11 studies were population-based studies [24].

### 4.2. Clinic-Based Studies of Episodic Headache and Psychological Comorbidities

Patients attending specialist headache clinics may represent a special group of patients who may include those with frequent, severe or difficult-to-treat headaches. These patients may be seen as a subset of children with headaches who may be on the severe end of the headache spectrum, but these studies are important as these children may have special characteristics that need clinical assessment and attention. Several studies have looked at the psychological comorbidities in this group, and different studies reached different conclusions about the risk of anxiety and depressive symptoms.

One of the earliest reports on the psychosocial factors in children with headache examined a large series of patients (migraine 151, TTH 94 and control children 96) in a clinical setting. Children with migraine, TTH and control had a similar socioeconomic background, similar family structure and school environment. However, children with TTH seemed to be more likely to have divorced parents and have fewer peer relations than patients with migraine and control children, but they were similar in other psychosocial factors [25].

A recently published meta-analysis of studies looking at psychopathological symptoms in children and adolescents with migraine and tension type-headache in clinical settings included 10 studies with reliable data [26]. The diagnosis of headache disorders was based on the International Classification of Headache Disorders (ICHD-2), and the assessment of psychopathological disorders was based on appropriate scoring on the internalising and externalising Child Behaviour Check List (CBCLi) and (CBCLe). This large meta-analysis reported on a combined patient population of 406 children with migraine, 230 with TTH and 488 control children matched for age, gender and intelligence quotient (IQ) [26]. Patients with migraine were shown to have a higher total CBCL score than controls, with a more marked effect in the CBCLi score (*p* < 0.001) than in the CBCLe (*p* > 0.001). Patients with TTH also exhibited a higher total CBCL score than control children, but CBCLi had a major effect on the total score (*p* = 0.001), more than that of CBCLe (*p* = 0.051). In comparing patients with migraine against patients with TTH, there were no significant differences between the two groups in total CBCL (*p* = 0.73), CBCLi (*p* = 0.954) or CBCLe (*p* = 0.649) [26].

On the other hand, a systematic review published two years earlier of seven studies, including four studies assessed in the above meta-analysis, looked at psychological functioning in children with migraine in the clinical setting. This systematic review showed serious problems and limitations due to the marked heterogeneity of the studies and the outcome measures. However, it concluded that children with migraine have a similar risk for withdrawn behaviour, thought problems, social problems, and delinquent or aggressive behaviour as control children. However, children with migraine have more somatic complaints and internalising behaviour [27].

Psychological symptoms in children of parents who suffer from recurrent headaches were shown to be similar to those in children with healthy parents who did not suffer from headache, suggesting an equal risk for psychological and family functioning in both groups [28].

## 5. Children with Chronic Daily Headache (CDH)

CDH is defined as a headache occurring on at least 15 days per month for at least three consecutive months. CDH is an umbrella label, and the underlying headache disorder can be one or a combination of CM, CTTH, Newly Presenting Daily Headache (NDPH), hemicrania continua, chronic cluster headache, medication overuse headache, persistent posttraumatic headache or other secondary headaches.

### 5.1. Population-Based Studies of Chronic Daily Headache and Psychological Comorbidities

Despite the high impact of chronic daily headaches on children’s quality of life and the associated disability, there is only a small number of population-based studies looking at psychological and other comorbidities in children with CDH.

A study in Taiwan identified 122 adolescents (12–15 years old) who had CDH from a population of 7900 adolescents giving a prevalence of 1.5% [29]. Eighty children had CTTH and eight had CM, while 24 overused medications. Detailed psychiatric assessment was completed on 121 children (90 female) with a mean age of 13.8 years. At least one psychiatric disorder was diagnosed in 57 adolescents (47%); 36 (30%) had depression, 43 (36%) had anxiety and 24 (20%) had a high suicidal risk score (>10) [30]. Female gender and older age were associated with depressive disorders. Migraine with aura was more strongly associated with psychological disorders than migraine without aura and independently predicted a high suicidal risk (adjusted OR = 6.0, *p* = 0.028) [30].

An epidemiological study of chronic daily headaches and CM in the USA in adolescents between 12 and 17 years of age estimated the prevalence of CM at 0.79% and 1.75% for CM with medication overuse headaches. Up to 90% of children with CM scored over 60 on HIT-6 and 75% scored over 17 on PedMIDAS, suggesting a severe disability [31].

### 5.2. Clinic-Studies of Children with Chronic Daily Headache

CDH in specialist clinics accounts for at least one in three of all patients referred for further assessment and management. Girls are more likely to be affected than boys, and the mean age of presentation is between 11 and 13 years [10,11].

Several studies looked at predisposing factors for the chronification of primary headache disorders and found some psychological factors and stressors to be contributing factors [11,32]. Other studies showed an increase in the frequency of headache attacks when patients encounter psychological stressors such as school failure, change, or family problems and illnesses [33].

Psychological and other comorbidities were reported in a large study of 115 patients with CDH (majority with CTTH) attending a specialist headache clinic. One in four patients had significant chronic neurological diseases (epilepsy, head injury, deafness, dysmorphic syndrome, corrected craniosynostosis, etc.) or psychological disorders (significant behavioural disorders and tics). One in ten patients had serious stressful life events related to family illnesses or recent bereavement [10].

A similar study in Canada looked at 143 children with CDH (majority with CTTH; 56%) and showed that stressors (as agreed upon by the child, the family and the attending physician) were likely contributing factors in CDH in 42% of patients. Stressors were described as related to school performance, bullying, peer pressure, parental separation or family illness/circumstances. Anxiety and depression were diagnosed in 6% and 9%, respectively [34].

Psychological comorbidities, including anxiety, sleep disorders and mood disturbances, were shown to affect two-thirds of 59 children (7–17 Years) with CDH, mainly CTTH, who were followed for up to four years [35].

## 6. Predictors of Psychological Comorbidities of Headache in Children and Young Adults

Management of children with headaches should take into account the presence of psychological problems and their interaction with the headache disorder. Assessment of children with headaches should also recognise factors that may predict the development of comorbid psychological problems (Table 1).

There is some evidence to suggest that early life events, health problems and behavioural characteristics may influence the development and predict the occurrence of headaches in childhood. A longitudinal study of over 1400 children from birth to age six years suggested that certain characteristics in a child’s life and health may predict the development of headaches at the age of six years [36]. These characteristics included an infant’s poor health and feeding problems at the age of nine months and sleeping difficulties at the age of three years. Headache in other family members, especially in the mother, predicted headaches in the preschool-age child. Travel sickness, nocturnal enuresis and the presence of long-term disease at the age of five years were strong predictors of later headaches. Also, at the age of five years, concentration difficulties, behavioural problems, unusual tiredness and, conversely, high sociability predicted headache [36].

In school-age children, it has been shown that a history of migraine in first-degree relatives and other pains and aches increase the risk and predict the occurrence of migraine [8,22]. However, the scores on the ‘anxious-depressed’ and ‘withdrawal’ subscales on the CBCL and the ‘externalising syndrome scale’ did not predict an increased risk for migraine or TTH, neither did the mean score of ‘total behaviour problems’ and ‘social competence scale’ [22].

In another longitudinal follow-up study, the occurrence of recurrent abdominal pain (RAP) in childhood predicted the development of psychological distress in young adults at age 18 years [37]. Experience with RAP over a short period of time significantly increased the risk for emotional distress disorders during young adulthood. Chronicity of RAP stretching over a longer period during childhood further increases the risk for later emotional distress disorders, depression and anxiety [37]. As there is a strong association between RAP and headaches in children, it is not unreasonable to deduce a similar risk for children with headaches [38,39].

A recent cross-sectional study compared children with primary headache and their mothers/caregivers to a matched control group and made an assessment of parents and children for emotional awareness using the Emotion Awareness Questionnaire, emotional experience using the Positive and Negative Affect Schedule and psychological adjustment using the Strength and Difficulties Questionnaire [40]. High headache frequency, regardless of the headache subtype, was closely related to the child’s emotional well-being. Also interestingly, the mother of children with high-frequency headaches had experienced less emotional clarity (DERS), and their children experienced overall more adjustment difficulties [40].

In recent meta-analyses of published studies, children and adolescents with migraine were shown to be more likely to have anxiety symptoms and disorders as well as depressive symptoms and disorders than healthy children and adolescents. Interestingly, the same results were seen in clinical and community/population-based samples, and there was no evidence of publication bias [41].

## 7. Predictors of the Persistence of Chronic Daily Headache

The natural course of headache in children is that of episodes of high-frequency headache episodes interspaced by periods of no or low-frequency headache. In some children, chronic daily headache may persist for years. Major depression and medication overuse were found to be strongly associated with the persistence of daily headaches in a follow-up study of 122 adolescents for three years [42].

In a large retrospective study of 5316 children and adolescents with headaches, the risk of worsening headache was significantly associated with increasing child’s age, female sex, chronic migraine, status migrainosus, depressive symptoms, higher PedMIDAS scores and use of nutraceuticals [43].

## 8. Summary and Conclusions

Affective disorders and primary headaches share common and complex pathophysiological pathways and clinical characteristics. The comorbidity of anxiety, depression, tension-type headache and migraine are frequently encountered in clinical practice and especially in children and adolescents attending specialist clinics with chronic daily headache. The relationship between headaches and psychological disorders is probably bidirectional, with one condition acting as a trigger to another. However, this relationship seems to be weaker in the general childhood population, as seen in population-based studies. Several factors may predict the increased risk of physical and psychological comorbidities, including female gender, highly frequent headache attacks, long history of chronic headaches, medication overuse and some personality traits.

Assessment of children with chronic headaches should include an assessment and management of possible psychological comorbidities in order to achieve optimal management of childhood headaches. The use of appropriate screening tools for anxiety and depression may allow early diagnosis, treatment and prevention of worsening of the daily headache.

## 9. Learning Points

- Headache and migraine are common in children and adolescents.
- Children with chronic migraine and frequent headaches are at increased risk for mood disorders, anxiety and depressive symptoms.
- Screening for psychological disorders in children with chronic daily headaches may improve management and improve outcomes.

## Figures and Tables

**Table 1:** Predictors of comorbid psychological disorders in children with primary headache disorders.

**Early Life events:**
Long-term illness in preschool-age children
Prolonged or repeated admissions to the hospital
Feeding and sleeping disorders in infants
**Events in school-age children:**
Separation anxiety
Behavioural and relationships problems
Psychosocial stressful experiences (chronic worries, social isolation etc.)
Recurrent pain disorders (abdominal, limb and musculoskeletal pain)
Attention Deficit and Hyperactivity Disorder
Autism Spectrum Disorder
**Other comorbid disorders:**
Other chronic pain syndromes
Long-term physical poor health
**Characteristics of headache disorders:**
Frequent episodic and chronic headache disorders
Inadequate treatment of headache
Headache leading to an adverse impact on children’s education, school attendance and quality of life, which in turn worsen the headache
**Family history:**
Parental or siblings’ long-term illnesses
Family history of chronic pain disorders, including headache
Maternal/caregiver with adverse emotional or psychological difficulties

## Data Availability

Not applicable.
